# Applications of the Behavior Change Wheel in promoting physical activity among children and adolescents: A scoping review

**DOI:** 10.1371/journal.pone.0354697

**Published:** 2026-07-31

**Authors:** Zheng Huang, Shuguang Fang, Sanying Peng

**Affiliations:** 1 Department of Physical Education, Changzhou Institute of Technology, Changzhou, China; 2 Department of Physical Education, Hohai University, Nanjing, China; Indiana University South Bend, UNITED STATES OF AMERICA

## Abstract

**Background:**

Physical inactivity among children and adolescents remains a major global public health concern, yet the ways in which behavioral theory is applied to inform physical activity (PA) interventions are often insufficiently described. The Behavior Change Wheel (BCW) model provides a systematic framework for linking behavioral determinants to intervention content, but its application in PA promotion for this population has not been comprehensively mapped.

**Methods:**

This scoping review was conducted in accordance with the Joanna Briggs Institute methodology and reported following the PRISMA-ScR guideline. Seven electronic databases were searched for studies published since 2011 that explicitly applied the BCW to promote PA among children and adolescents. Data were charted on study characteristics, targeted components of the Capability, Opportunity, and Motivation-Behavior (COM-B) model, BCW intervention functions, behavior change techniques, and implementation outcomes.

**Results:**

Fifteen studies met the inclusion criteria. Most applications focused on intervention development, co-design, or feasibility rather than effectiveness evaluation. Psychological capability and reflective motivation were the most frequently targeted COM-B components, and education, training, and enablement were the dominant intervention functions. Commonly used behavior change techniques included goal setting, action planning, self-monitoring, feedback, and social support. Functions involving coercion or restriction were rarely used. Reporting of implementation outcomes was inconsistent, with limited attention to reach, penetration, and sustainability.

**Conclusions:**

In studies using the BCW framework, PA interventions for children and adolescents predominantly emphasize individual-level self-regulation strategies, with less focus on opportunity-level change and implementation sustainability. Greater transparency in theoretical mapping and stronger integration of implementation considerations are needed to enhance the translation of theory-based interventions into practice.

## 1 Introduction

Physical inactivity among children and adolescents has become a persistent global public health concern rather than a transient lifestyle issue. Worldwide surveillance indicates that approximately 81% of adolescents aged 11–17 years fail to achieve the World Health Organization (WHO) recommendation of at least 60 minutes of moderate-to-vigorous physical activity (MVPA) daily [1,2]. Despite sustained policy attention and large-scale school- and community-based initiatives, population-level physical activity (PA) levels in children and adolescents have shown little improvement over the past decade [2,3]. This pattern is concerning given the well-established associations between insufficient PA and adverse cardiometabolic, mental health, and cognitive outcomes in childhood and adolescence [3–5]. Moreover, PA behaviors track strongly from adolescence into adulthood, contributing to the long-term burden of non-communicable diseases across the life course [5,6].

In response, numerous PA promotion interventions targeting children and adolescents have been developed. However, recent systematic reviews and meta-analyses consistently report small and heterogeneous effects, with limited evidence of sustained behavior change [3,4,7]. One frequently cited explanation is the limited use of behavioral theory in intervention design and reporting [8]. Many interventions focus on educational or environmental components without clearly articulating the behavioral determinants they aim to modify or the mechanisms through which change is expected to occur [7,9]. This “black box” approach constrains cumulative learning, impedes replication, and weakens translation into practice.

Contemporary guidance for complex interventions, including that from the UK Medical Research Council, emphasizes the importance of theory-informed development to support more transparent intervention development, evaluation, and interpretation [10,11]. Within this context, the Behavior Change Wheel (BCW) has emerged as a widely used integrative framework for intervention design [12]. The BCW is underpinned by the Capability, Opportunity, and Motivation-Behavior (COM-B) model as its core, which frames behavior as a function of capability, opportunity, and motivation. More specifically, the BCW is organized into three interrelated layers: at the center is the COM-B model, which provides a framework for behavioral diagnosis; surrounding this are nine intervention functions that guide the selection of broad strategies for changing behavior; and the outer layer comprises policy categories that can support intervention delivery and wider implementation [12]. This structure supports a systematic translation from behavioral diagnosis to the selection of intervention functions and the specification of intervention content using behavior change techniques (BCTs) [12,13]. By linking behavioral analysis to intervention planning in this way, the BCW helps make the theoretical basis of an intervention more explicit and facilitates more transparent reporting of how intervention content is derived from identified determinants of behavior [12]. Over the past decade, the BCW has been applied across a range of health behaviors and settings, including smoking cessation, diet, diabetes management, antimicrobial stewardship, and PA promotion [14–17]. This may be especially relevant for PA promotion among children and adolescents, as PA behavior in these populations is shaped by interacting individual, interpersonal, institutional, and environmental factors.

Nevertheless, important gaps remain in how the BCW has been applied to PA promotion among children and adolescents. This population’s PA is developmentally contingent and strongly conditioned by structural opportunity, including school routines, parental support, and neighborhood resources. These features complicate behavioral diagnosis and raise questions about whether COM-B constructs are being operationalized in ways that reflect the realities of this population’s settings and decision-making [9,18]. At the same time, there is a recurring concern that the BCW is sometimes invoked more as a marker of rigor than as a framework that is used systematically across the full design pathway, a practice often described as theoretical tokenism [19]. Where this occurs, the mapping process is frequently left opaque. In particular, the linkages from COM-B components to BCW intervention functions and then to specific BCTs are not consistently reported, making it difficult to identify the active ingredients and to accumulate learning across studies [17,19]. Moreover, the literature continues to privilege effectiveness outcomes, while reporting of implementation processes such as feasibility, acceptability, and fidelity remains limited, which constrains inference about how BCW-informed interventions are implemented, reported, and potentially sustained in real-world youth contexts [20,21].

To date, no scoping review has systematically mapped how the BCW has been applied to promote PA among children and adolescents, nor examined the transparency of theoretical mapping and reporting in this field. A scoping review is particularly appropriate for this purpose because the key question is not whether BCW-informed interventions are more effective than other theory-based interventions, but what is known about how the BCW and COM-B model have been used, operationalized, and reported in this area. Addressing this gap is critical for strengthening theory-informed PA interventions and supporting clearer reporting, replication, and future intervention development. Therefore, this scoping review aims to: (1) map the extent and characteristics of BCW applications in PA promotion among this population; (2) examine how behavioral determinants, intervention functions, and BCTs are selected and reported; and (3) summarize reported implementation outcomes and identify gaps in theoretical mapping and reporting practices. By clarifying how behavioral theory is currently operationalized, this review seeks to inform future theory-informed development and reporting of PA interventions for children and adolescents.

## 2. Methods

### 2.1 Protocol and registration

This scoping review was conducted in accordance with the Joanna Briggs Institute (JBI) methodology for scoping reviews [22] and is reported following the Preferred Reporting Items for Systematic Reviews and Meta-Analyses extension for Scoping Reviews (PRISMA-ScR) guideline [23].

A detailed review protocol specifying the review objectives, eligibility criteria, information sources, and planned methods was developed a priori and registered in the International Prospective Register of Systematic Reviews (PROSPERO) (registration number: CRD420251130981). The protocol was finalized prior to the commencement of study selection.

Any deviations from the registered protocol were documented and are reported transparently in the Methods or Results sections where applicable.

### 2.2 Eligibility criteria

Eligibility criteria were defined a priori using the Population–Concept–Context (PCC) framework recommended for scoping reviews [22].

Population. Studies targeting children and adolescents aged 0–18 years were eligible. Studies involving additional stakeholders (e.g., parents, teachers, coaches, healthcare professionals) were included if the intervention or analysis was explicitly designed to promote PA in children or adolescents.

Concept. Studies were required to explicitly report the use of the BCW or the COM-B model [12]. Eligible applications included, but were not limited to, intervention development, behavioral diagnosis (e.g., identification of barriers and facilitators), content mapping, process evaluation, and measurement development. Studies using the Theoretical Domains Framework (TDF) were included only when TDF was applied in conjunction with BCW or COM-B, reflecting the integrated nature of these frameworks [12,13].

Context. All settings were eligible, including schools, community environments, home settings, healthcare or rehabilitation services, and digital or online platforms. No geographical restrictions were applied.

Types of evidence. Empirical studies of any design were eligible, including quantitative, qualitative, and mixed-methods studies. This encompassed intervention development studies, feasibility and pilot trials, process evaluations, and measurement or instrument development studies.

Exclusion criteria. Editorials, opinion pieces, commentaries, and conference abstracts without full empirical data were excluded.

### 2.3 Information sources and search strategy

A comprehensive literature search was conducted across seven electronic databases: PubMed/MEDLINE, Embase (Ovid), PsycINFO (EBSCOhost), CINAHL (EBSCOhost), Scopus, Web of Science Core Collection, and the Cochrane Library (Wiley). The search strategy was developed in consultation with an experienced research librarian and combined controlled vocabulary (e.g., MeSH terms) with free-text terms relating to “Behavior Change Wheel,” “COM-B,” “physical activity,” and separate age-related terms such as “children” and “adolescents.” The search covered peer-reviewed empirical studies published in academic journals from 1 January 2011, corresponding to the initial publication of the BCW framework [12], to the date of the final search. Grey literature, including government reports, conference abstracts, and theses/dissertations, was not searched or included. This decision was made because the purpose of this review was to examine how the BCW and COM-B model were applied and reported in intervention research, particularly the transparency of theoretical mapping from behavioral diagnosis to intervention functions and BCTs. Such information is often inconsistently reported or unavailable in grey literature, which would limit the feasibility of applying the same extraction and coding criteria across sources. No language restrictions were applied. Where non-English records were retrieved, screening and data extraction were conducted using bilingual reviewers or validated machine translation tools. Detailed search strategies for all databases are presented in S1 File. To enhance the completeness of the search within the published peer-reviewed literature, reference lists of included studies and relevant reviews were manually screened to identify additional eligible records.

### 2.4 Selection of sources of evidence

All identified records were imported into reference management software (EndNote 20) for screening and deduplication. Study selection was conducted in two stages. First, titles and abstracts were independently screened by two reviewers against the predefined eligibility criteria. Second, full texts of potentially relevant records were retrieved and independently assessed by the same reviewers. Disagreements at either stage were resolved through discussion, with consultation of a third reviewer when consensus could not be reached. Prior to formal screening, a pilot calibration exercise was conducted on a subset of records to ensure consistent interpretation of eligibility criteria, with a target Cohen’s kappa ≥ 0.70, indicating substantial inter-rater agreement [24]. The overall study selection process is reported using a PRISMA-ScR flow diagram.

### 2.5 Data charting process

Data were extracted using a standardized data charting form developed specifically for this review and implemented in a structured Microsoft Excel spreadsheet, which had been refined through prior empirical use to ensure efficiency and consistency. The form was pilot-tested on a subset of included studies and iteratively refined to enhance clarity and completeness. Two reviewers independently charted data from each included study, with discrepancies resolved through discussion and consensus. The charting process captured key study characteristics (authors, year of publication, country, study design, setting, and target population), details of theoretical application (targeted COM-B components, selected BCW intervention functions, and reported BCTs), and information on implementation and feasibility. Where BCTs were reported using different taxonomies or classification systems, these were mapped and converted to equivalent categories within the BCT Taxonomy v1 to enable consistent synthesis, as detailed in S2 File.

Implementation-related data included reported outcomes based on Proctor et al.’s implementation outcomes framework (e.g., acceptability, adoption, feasibility, fidelity, reach, and sustainability), as well as trial feasibility indicators such as recruitment and retention rates where available [21].

### 2.6 Critical appraisal

Consistent with methodological guidance for scoping reviews, a formal assessment of methodological quality or risk of bias was not undertaken [22,23]. The primary purpose of this review was to map the extent, nature, and characteristics of BCW and COM-B application rather than to evaluate intervention effectiveness. The transparency of theoretical reporting was assessed descriptively by examining whether explicit links between COM-B components, BCW intervention functions, and BCTs were reported, and the findings are presented in the Results.

### 2.7 Synthesis of results

Extracted data were synthesized using descriptive statistics and narrative synthesis. Frequencies and distributions of targeted COM-B components, BCW intervention functions, and BCTs were summarized in tables and visualized using bar charts to illustrate patterns across studies and settings. A narrative synthesis was undertaken to contextualize these quantitative findings, with particular attention to identifying recurring patterns, gaps in theoretical mapping, and inconsistencies in implementation outcome reporting across populations, settings, and study designs.

## 3 Results

### 3.1 Selection of sources of evidence

**Fig 1:**
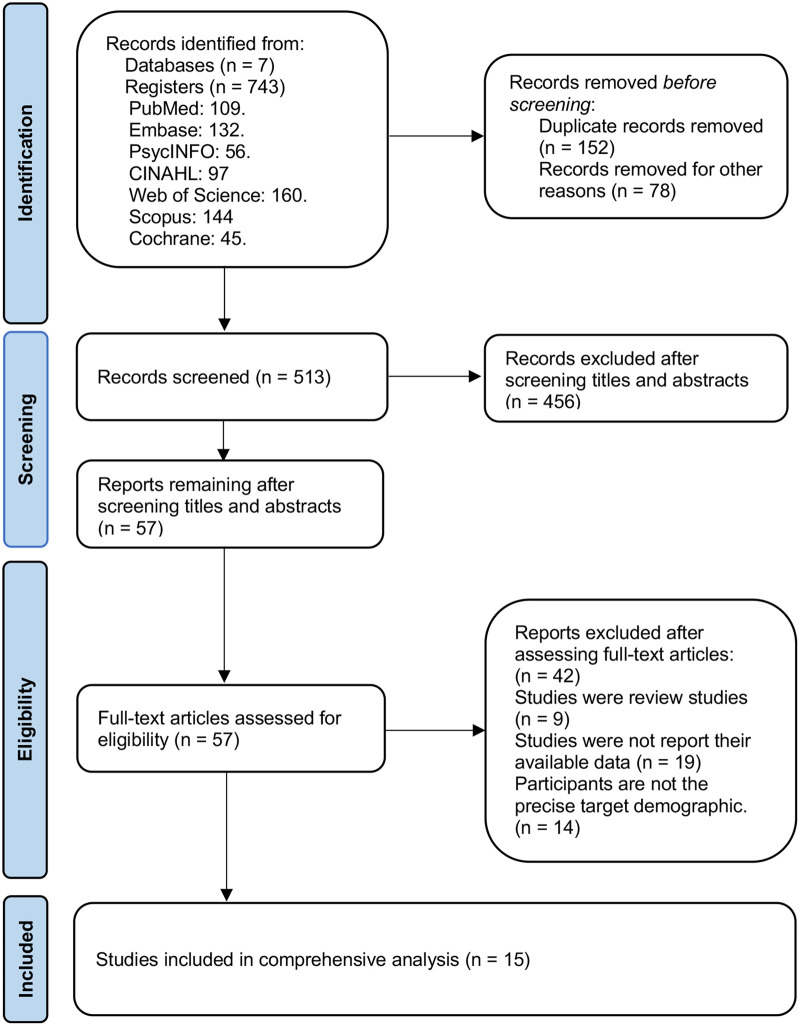
Flowchart of the literature search process.

The study selection process is illustrated in Fig 1. Database searches identified 743 records. After removal of duplicates (n = 152) and records with insufficient bibliographic information (n = 78), 513 records remained for title and abstract screening. Of these, 456 records were excluded as clearly not meeting the inclusion criteria. Fifty-seven full-text articles were assessed for eligibility. Following full-text review, 42 articles were excluded, primarily because they were review papers (n = 9), did not report relevant empirical data (n = 19), or did not involve the target population of children or adolescents (n = 14). Ultimately, 15 studies met all eligibility criteria and were included in the scoping review [18,25–38].

### 3.2 Characteristics of included studies

The key characteristics of the included studies are summarized in Table 1. The studies were published between 2015 and 2025 and were conducted predominantly in Ireland [18,25,27,32–34], followed by the United Kingdom [28,29,36], Australia [30,35], China [37,38], the United States [26], and Belgium [31].

**Table 1:** Characteristics of included studies.

Study	Setting	Country/Region	Participant: mean age (SD), sample size	PA measurement method	PA outcome(s)	Digital/wearables used	Notes/decisions
Brennan et al., 2025	Suburban primary school; Digital Online	Ireland	Girls: 10–12, 10; Mothers and female guardians: NR, 9; Teachers: NR, 6	NR	None (development of prototype; no effectiveness outcomes)	Y	Three-phase co-design targeting preteen girls (10–12y) of low SEP and their mothers; BCW functions and BCTs specified; prototype app developed; ready for feasibility.
Caru et al., 2024	Healthcare/Clinical; Home-based remote	USA	Survivors of childhood cancer: 13.6, 40	Combined	6-minute walk test (improved vs control); Fitbit steps; % meeting PA guidelines	Y	12-week tailored home PA program with weekly support calls; Fitbit Inspire 2; high acceptability/feasibility; estimated cost per participant ~ $126.57; no serious AEs.
Corr & Murtagh, 2020	Post-primary school.	Ireland	Adolescent girls: 15–17, 31	Combined	Steps (pedometer; incomplete data); PAQ-A; enjoyment; feasibility benchmarks met	Y	Post-primary school PE setting; 6-week co-created sessions; restriction/env. restructuring excluded in BCW selection; high attendance/retention.
Creaser et al., 2023	Community; Digital/Online	UK	Children: 8 (4.01), 12; Parents: 42 (5.68), 11	NR	None (development study; intervention specified with components)	Y	Family-based wearable program (Fitbit Alta HR) with eight components; experts + families co-design; six BCW functions used; 24 BCTs enumerated.
Faghy et al., 2021	School	UK	School-age children: 8.9 (1.3), 147	Combined	PA frequency (no change); sedentary time & MVPA (no significant change); fruit/veg intake improved	Y	12-week school program (7–11y); questionnaire + accelerometer (subsample); used COM-B concepts to inform content; no explicit BCW/BCT mapping reported.
Grimshaw et al., 2022	Tertiary pediatric hospital oncology service; intervention development workshops	Australia	School-aged children: 5–16, NR	NR	NR (development paper)	Y	Development paper that precedes feasibility testing of the same CanMOVE program
Maenhout et al., 2024	Secondary education	Belgium	Adolescents with mild intellectual disabilities: 14.22 (0.44), 9	NR	NR (development paper)	Y	Development stage preceding planned feasibility evaluation
Martin & Murtagh, 2015	Primary school classrooms	Ireland	Students from primary schools: NR, >200; Teacher: NR, NR	Objective	Primary outcome: minutes of MVPA during school time via ActiGraph accelerometers at baseline, post (8 weeks), and 4-month follow-up	NR	Design/protocol for subsequent cluster RCT evaluating the intervention
McDermott et al., 2022	School/community contexts (as per qualitative interviews)	Northern Ireland	Students with mild/moderate intellectual disabilities: 11–17, 7; Parents: NR, 12; Teacher: NR, 9	NR	Qualitative; no PA outcomes quantified	NR	Standalone qualitative diagnostic study to inform future interventions
McQuinn et al., 2022	School (post-primary, after-school peer-led classes)	Ireland	Adolescent females: 14.82 (1.71), 287; Teacher: NR, 7	Subjective	PACE+ questionnaire (baseline descriptive only); no outcome trial reported in this paper	Y	Outputs feed the registered feasibility trial
Murtagh et al., 2018	Community / family	Ireland	Adolescent girls: NR, NR; Mothers: NR, NR	NR	Not applicable (development paper)	NR	Designed content to be evaluated in future studies
Reedman et al., 2021	Home and community; physiotherapist-delivered weekly sessions	Australia	Children with cerebral palsy: 10 (1.6), 37	NR	This paper does not report outcomes; primary trial used COPM goal performance/satisfaction (context)	Y	Maps active ingredients and mechanisms underpinning prior effectiveness
Taylor et al., 2015	Residential weight‑management camp (UK); Schools (Spain)	UK; Spain	Obese children from England: 12.24 (2.01), 71; Obese children from Spain: 10.52 (1.23), 45	Subjective	No PA behavior outcomes; BMI SDS; PEC questionnaire pre/post (camp)	NR	Instrument may inform future COM‑B‑based interventions
Wang et al., 2021	School, family, and community (three-level program)	China	Chinese children: NR, NR	NR	To be assessed in later feasibility/definitive trials (not in this paper)	Y	First stage before feasibility non-RCT and process evaluation
Wang et al., 2022	Two public schools; 16‑week program; interviews conducted post‑intervention	China	Children: 11.2, 20; Parents: NR, 20Teacher: NR, 2	NR	Process evaluation only (qualitative); quantitative feasibility parameters reported elsewhere	Y	Qualitative complement to feasibility metrics to optimize future trial

Notes: BCT, behavior change technique; BCW, Behavior Change Wheel; BMI, body mass index; COM-B, Capability, Opportunity, Motivation-Behavior model; COPM, Canadian Occupational Performance Measure; HR, heart rate; MVPA, moderate-to-vigorous physical activity; NR, not reported; PA, physical activity; PACE + , Physically Active Children in Education Plus (study-specific questionnaire/measure); PAQ-A, Physical Activity Questionnaire for Adolescents; PE, physical education; PEC, Pediatric Energy Cost questionnaire; RCT, randomized controlled trial; SD, standard deviation; SDS, standard deviation score; SEP, socioeconomic position; UK, United Kingdom; USA, United States of America; Y, yes (reported).

Most interventions were implemented in school-based settings [18,25,27,29,31–33,35–38], often supplemented by family [26,34,37], community [28,33–35,37], or digital components [25,26,28]. Several studies targeted specific clinical or vulnerable populations, including childhood cancer survivors [26], children with cerebral palsy [35], and adolescents with intellectual disabilities [31,33], while others focused on general school-aged populations [18,25,27–30,32,34,36–38].

In terms of study design, a substantial proportion of the literature focused on intervention development, co-design, feasibility, or protocol reporting, rather than definitive effectiveness trials [18,25,30–32,34,37,38]. Accordingly, PA outcomes were heterogeneous. Some studies employed objective or combined measurement approaches (e.g., accelerometry, pedometers) [26,27,29,32], whereas others relied on self-report measures or did not report behavioral outcomes due to their developmental focus [18,25,28,30,31,33–38].

The use of digital technologies and wearable devices (e.g., Fitbit-based interventions) was common [18,25–31,35,37,38], reflecting a growing emphasis on technology-supported behavior change in PA interventions of children and adolescents.

### 3.3 Applications of the behavior change wheel and COM-B frameworks

The roles and modes of application of the BCW and COM-B frameworks are detailed in Table 2. Table 2 summarizes how the BCW and COM-B frameworks were applied across studies, including their application role, research stage, the Theory, Model, and Framework Comparison and Selection Tool (T-CaST) level, mapping transparency from COM-B to BCW and BCTs, and co-frameworks used. T-CaST is an implementation science tool for comparing and selecting theories, models, and frameworks. Across the included studies, these frameworks were used predominantly to inform intervention design and development, particularly within co-design and feasibility-focused research. A smaller number of studies applied COM-B for determinant analysis or measurement development.

**Table 2:** Use of the behavior change wheel and COM-B.

Study	Application role (BCW/COM-B)	Stage	T-CaST level	Mapping transparency (COM-B → BCW → BCT)	Co-frameworks used
Brennan et al., 2025	Design (intervention development/co-design)	Development	L3	Y	TDF; MRC framework; BCT Ontology; BCIO annotation
Caru et al., 2024	Design (intervention development/co-design)	Feasibility/Pilot	L3	N	ORBIT model; Pediatric oncology exercise guidelines
Corr & Murtagh, 2020	Design (intervention development/co-design)	Feasibility/Pilot	L3	Y	APEASE criteria; CONSORT/COREQ reporting
Creaser et al., 2023	Design (intervention development/co-design)	Development	L3	Y	TDF within BCW; co-design methods
Faghy et al., 2021	Measurement/tool development	Effectiveness/Trial	L2	N	COM-B and TDF
Grimshaw et al., 2022	Design (intervention development/co-design)	Development	L3	Y	COM-B and CALO-RE
Maenhout et al., 2024	Design (intervention development/co-design)	Development	L3	Y	COM-B and TDF
Martin & Murtagh, 2015	Design (intervention development/co-design)	Effectiveness/Trial	L3	Y	BCW
McDermott et al., 2022	Determinant analysis (barriers/facilitators via COM-B)	Process evaluation	L3	Y	BCW
McQuinn et al., 2022	Design (intervention development/co-design)	Development	L3	Y	BCW + COM-B + TDF + BCTTv1
Murtagh et al., 2018	Design (intervention development/co-design)	Development	L3	Y	BCW + COM-B + BCTTv1
Reedman et al., 2021	BCT mapping/evaluation (content coding)	Effectiveness/Trial	L3	Y	BCW + COM-B + TDF + BCTTv1 + ICF
Taylor et al., 2015	Measurement/tool development	Development	L3	Y	COM‑B (with MINDSPACE informing item generation)
Wang et al., 2021	Design (intervention development/co-design)	Development	L3	Y	BCW and TDF
Wang et al., 2022	Design (intervention development/co-design)	Feasibility/Pilot	L1	N	BCW and TDF

Notes: APEASE, Affordability, Practicability, Effectiveness/cost-effectiveness, Acceptability, Side-effects/safety, Equity; BCT, behavior change technique; BCT Ontology, Behavior Change Technique Ontology; BCTTv1, Behavior Change Technique Taxonomy v1; BCIO, Behavior Change Intervention Ontology; BCW, Behavior Change Wheel; CALO-RE, Coventry, Aberdeen & London–Refined taxonomy of behavior change techniques; COM-B, Capability, Opportunity, Motivation-Behavior model; CONSORT, Consolidated Standards of Reporting Trials; COREQ, Consolidated Criteria for Reporting Qualitative Research; ICF, International Classification of Functioning, Disability and Health; L1/L2/L3, T-CaST level 1/2/3; MINDSPACE, Messenger, Incentives, Norms, Defaults, Salience, Priming, Affect, Commitments, Ego; MRC, UK Medical Research Council; ORBIT, Obesity-Related Behavioral Intervention Trials (framework); T-CaST, Theory, Model, and Framework Comparison and Selection Tool; TDF, Theoretical Domains Framework; Y, yes (reported); N, no (not reported).

#### 3.3.1 Role of frameworks and mapping transparency.

Most studies demonstrated high levels of theoretical integration, explicitly linking COM-B components to BCW intervention functions and, in turn, to specific BCTs. However, several studies reported partial or implicit use of the frameworks, with limited transparency regarding the mapping process.

#### 3.3.2 Use of Co-frameworks.

The BCW and COM-B were frequently combined with other frameworks to enhance analytical depth. The TDF was the most commonly used supplementary framework, followed by the MRC framework for complex interventions, the CALO-RE taxonomy, and evaluative frameworks such as APEASE. This multimodal theoretical approach was particularly evident in studies focused on intervention development and optimization.

### 3.4 Theoretical mapping of behavioral determinants and intervention functions

**Fig 2:**
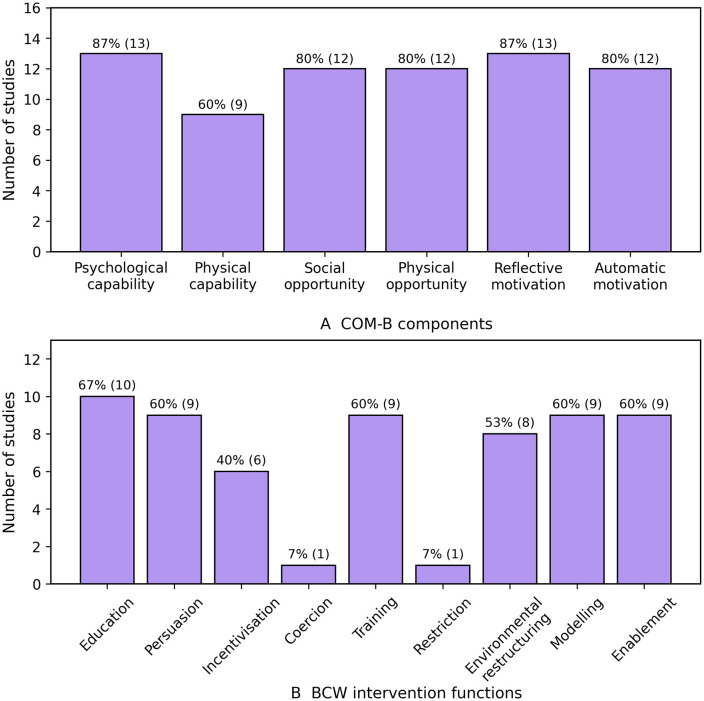
Distribution of targeted COM-B components and BCW intervention functions.

The distribution of targeted COM-B components and BCW intervention functions is presented in Fig 2, with detailed mappings provided in S3 File.

#### 3.4.1 Targeting COM-B components.

All six COM-B components were addressed across the included studies. Psychological capability and reflective motivation were the most frequently targeted determinants, appearing in 87% of studies, indicating a strong emphasis on knowledge, beliefs, and intentional processes related to PA. Physical capability and automatic motivation were targeted in 80% of studies, reflecting the importance of skill development, habit formation, and affective processes in PA promotion of this population. Social and physical opportunity were also frequently addressed (80%), underscoring the role of environmental and social contexts, particularly within school and family settings.

#### 3.4.2 Distribution of BCW intervention functions.

As shown in Fig 2B, the selection of BCW intervention functions was highly concentrated. Education, persuasion, training, modelling, environmental restructuring, and enablement were the most frequently employed functions. These functions aligned closely with the dominant COM-B determinants identified across studies.

In contrast, restriction and coercion were rarely used (7%), and in some cases explicitly excluded, suggesting a consistent preference for autonomy-supportive and developmentally appropriate strategies in interventions targeting children and adolescents.

### 3.5 Intervention content: Behavior change techniques

**Fig 3:**
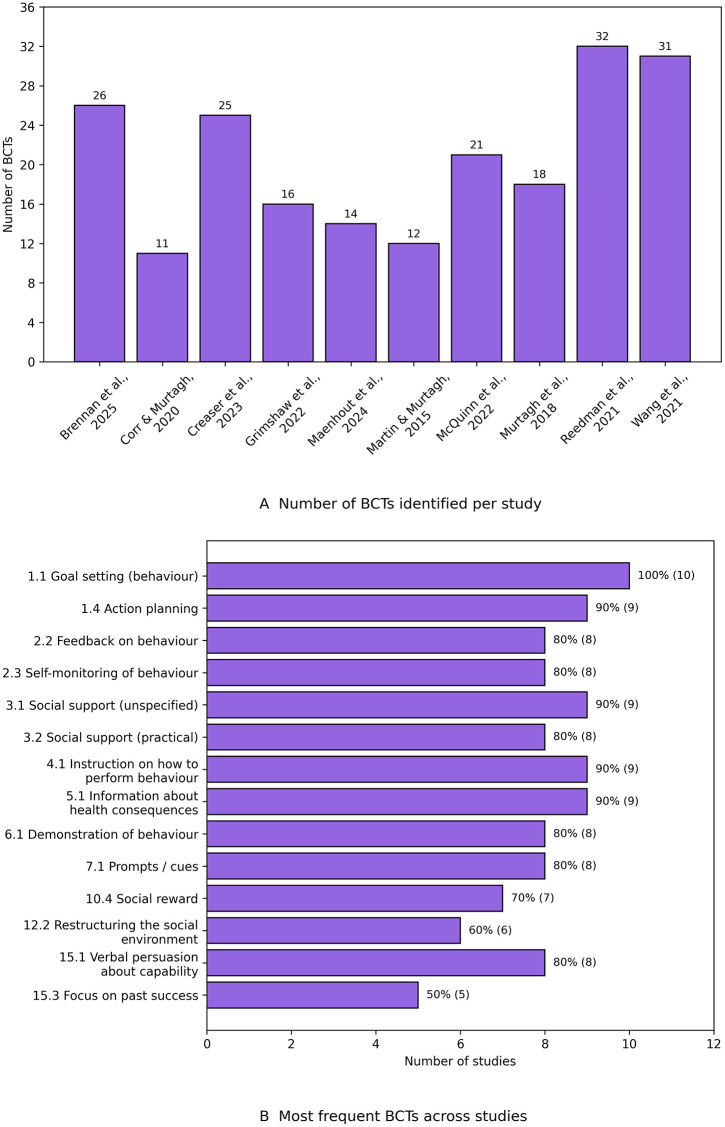
Mapping the applications of behavior change techniques.

The BCTs used across interventions are summarized in Table 3 and illustrated in Fig 3. Table 3 summarizes the BCT-related information reported across studies, including whether BCT mapping was presented, the taxonomy used, the number of BCTs identified, the main BCT clusters, which refer to the 16 higher-order groupings of BCT Taxonomy version 1 (BCTTv1), and the specific BCTs reported.

**Table 3:** Applications of behavior change techniques.

Study	BCT mapping present	BCT Taxonomy	BCT count (numeric)	Main BCT clusters	BCTs
Brennan et al., 2025	Y	BCIO	26	1; 2; 3; 4; 5; 6; 8; 9; 10; 11; 12; 13; 15	1.1; 1.4; 2.2; 2.3; 3.1; 3.2; 4.1; 5.1; 5.3; 5.4; 6.1; 6.2; 8.1; 8.2; 8.3; 8.7; 9.1; 10.4; 11.2; 12.5; 13.1; 13.2; 13.4; 15.1; 15.3; 15.4
Caru et al., 2024	NR				
Corr & Murtagh, 2020	Y	BCTTv1	11	1; 2; 4; 5; 6; 8; 9	1.1; 1.4; 2.2; 2.3; 2.6; 4.1; 5.1; 5.4; 6.1; 8.3; 9.1
Creaser et al., 2023	Y	BCTTv1	25	1; 2; 3; 4; 5; 6; 7; 8; 9; 10; 14	1.1; 1.3; 1.4; 1.5; 1.6; 1.7; 2.2; 2.3; 2.4; 2.7; 3.1; 3.2; 4.1; 5.1; 5.3; 5.6; 6.1; 6.2; 7.1; 8.7; 9.1; 10.3; 10.6; 10.10; 14.5
Faghy et al., 2021	NR				
Grimshaw et al., 2022	Y	CALO-RE BCT	16	1; 2; 3; 5; 6; 7; 10; 12; 15	1.1; 1.2; 1.4; 1.5; 2.2; 2.3; 3.1; 3.2; 5.1; 6.1; 7.1; 10.4; 10.9; 12.1; 12.2; 15.3
Maenhout et al., 2024	Y	BCTTv1	14	1; 2; 3; 5; 6; 9; 10; 12; 15	1.1; 1.4; 1.5; 2.2; 3.1; 3.2; 3.3; 5.1; 5.2; 6.1; 9.1; 10.4; 12.2; 15.1
Martin & Murtagh, 2015	Y	BCTTv1	12	1; 2; 4; 5; 7; 8; 9; 12	1.1; 1.2; 1.4; 2.3; 4.1; 5.3; 7.1; 8.2; 8.4; 8.6; 9.1; 12.1
McDermott et al., 2022	NR				
McQuinn et al., 2022	Y	BCTTv1	21	1; 2; 3; 4; 5; 6; 7; 8; 9; 10; 12; 15	1.1; 1.4; 2.1; 3.2; 3.3; 4.1; 5.1; 5.4; 6.1; 7.1; 8.1; 8.3; 8.6; 9.1; 10.1; 10.2; 10.3; 10.4; 10.6; 12.2; 15.1
Murtagh et al., 2018	Y	BCTTv1	18	1; 2; 3; 4; 5; 6; 7; 9; 10; 13; 15	1.1; 1.2; 1.4; 1.5; 2.2; 2.3; 3.1; 3.2; 4.1; 5.1; 5.3; 5.6; 6.1; 7.1; 9.1; 10.4; 13.1; 15.1
Reedman et al., 2021	Y	BCTTv1	32	1; 2; 3; 4; 6; 8; 9; 12; 13; 15	1.1; 1.2; 1.4; 1.5; 1.6; 1.8; 1.9; 2.1; 2.2; 3.1; 3.2; 3.3; 4.1; 6.1; 8.1; 8.2; 8.3; 8.6; 8.7; 9.1; 9.2; 9.3; 12.2; 12.5; 12.6; 13.1; 13.2; 13.3; 13.4; 15.1; 15.3; 15.4
Taylor et al., 2015	NR				
Wang et al., 2021	Y	BCTTv1	31	1; 2; 3; 4; 5; 6; 7; 8; 9; 10; 12; 13; 15	1.1; 1.2; 1.3; 1.4; 1.5; 1.7; 2.1; 2.2; 2.3; 2.5; 2.7; 3.1; 3.2; 3.3; 4.1; 5.1; 5.2; 5.3; 6.1; 7.1; 8.1; 8.7; 9.1; 9.2; 10.2; 10.4; 12.1; 12.5; 13.1; 15.1; 15.3
Wang et al., 2022	NR				

Notes: BCIO, Behavior Change Intervention Ontology; BCT, behavior change technique; BCT count, total number of distinct BCTs identified in the study; BCT clusters, higher-level groupings of BCTs according to the Behavior Change Technique Taxonomy; BCTs, specific behavior change techniques coded using numeric labels (e.g., 1.1, 2.2); BCTTv1, Behavior Change Technique Taxonomy version 1; CALO-RE, Coventry, Aberdeen & London–Refined taxonomy of behavior change techniques; NR, not reported; Y, yes (reported).

#### 3.5.1 Volume and taxonomy of behavior change techniques.

Among studies that reported explicit BCT mapping, the number of techniques ranged from 11 to 32 per intervention. The majority employed BCT Taxonomy v1 (BCTTv1), facilitating standardized reporting and comparison across studies. A smaller number of studies used earlier taxonomies or ontology-based classifications.

#### 3.5.2 Most frequent behavior change techniques.

As shown in Fig 3B, Goal setting (behavior) [BCT 1.1] was the most consistently applied technique, appearing in 100% of mapped studies (n = 10). A cluster of BCTs was used in 90% of studies, including Action planning (1.4), Social support (unspecified) (3.1), Information about health consequences (5.1), and Instruction on how to perform the behavior (4.1). Additional techniques appearing in ≥80% of studies included Self-monitoring of behavior (2.3), Feedback on behavior (2.2), Demonstration of behavior (6.1), Prompts/cues (7.1), and Verbal persuasion about capability (15.1). Overall, intervention content was characterized by a strong emphasis on self-regulation strategies, complemented by instructional and social support components.

### 3.6 Reporting of implementation outcomes

Implementation outcomes were inconsistently reported across studies (shown in S4 File). Acceptability and feasibility were the most frequently assessed outcomes, particularly within feasibility and pilot studies. In contrast, key implementation dimensions, including adoption, fidelity, reach, and sustainability, were seldom documented.

Only one study reported implementation cost, and no study provided comprehensive coverage across multiple implementation outcomes. This pattern highlights a substantial gap in the evaluation of real-world applicability and scalability of BCW-informed PA interventions for children and adolescents.

## 4. Discussion

This scoping review mapped the extent and nature of applications of the BCW framework in PA promotion research for children and adolescents. The included evidence base remains relatively small and skewed toward intervention development, co-design, feasibility and diagnostic work, rather than fully powered effectiveness trials. This pattern is not necessarily a weakness, as recent guidance on complex interventions emphasizes that iterative development, explicit theorization, and early contextual attention are essential prerequisites for large-scale evaluation and scale-up [11].

A distinct “theory-to-technique” profile emerged. In studies offering sufficient detail, intervention content predominantly focused on capability building and self-regulation through strategies such as goal setting, action planning, instruction, feedback, and practical social support. Such clustering is broadly consistent with the wider PA and digital behavior change literature, where planning/monitoring and instructional components are frequently deployed as the core active ingredients [31,39,40]. Although some interventions incorporated interpersonal elements such as practical social support, their overall emphasis remained primarily on capability building and student-level self-regulation. School-based PA scholarship increasingly suggests that such approaches alone rarely shift overall PA levels sustainably because PA opportunities among children and adolescents are structurally bounded by timetables, staff capacity, facilities, safety norms, and competing educational priorities [41–43]. In this sense, the mapped BCT profile may reflect both a strength (a coherent, replicable toolkit) and a potential constraint (a tendency to default to student-level self-regulation even when opportunity constraints are dominant). The practical implication is that the BCW should be utilized not only to justify the selection of specific techniques, but also to ensure that intervention functions effectively address the primary constraints on this population’s PA, which frequently involve physical and social opportunities within school and community settings.

A particularly significant yet overlooked finding in this review is the minimal reliance on BCW functions centered on control or punishment. Specifically, coercion [27] and restriction [30] both appeared in only 7% of studies, meaning that these intervention functions were included in only a few intervention designs and were not used in most studies. This is notable because it may reflect a preference for non-coercive and autonomy-supportive approaches in PA promotion for children and adolescents [44]. The BCW explicitly includes coercion and restriction as potential intervention functions, but their use carries practical and normative risks in this population’s settings (e.g., undermining autonomy, reducing enjoyment, and provoking resistance) [12]. The broader motivational literature supports this interpretation: autonomy-supportive climates are consistently associated with more adaptive motivational profiles and higher PA engagement in adolescents, whereas controlling approaches tend to predict poorer motivation and disengagement [45–47]. For practitioners, this finding reinforces a concrete design principle: youth PA interventions should prioritize positive, competence-building, autonomy-supportive strategies (e.g., choice, mastery-oriented goals, supportive feedback, peer modelling, enjoyable routines), rather than punitive contingencies or restrictive controls. Evidence from self-determination theory-based research indicates that PA intervention effects are often driven by enhanced autonomous motivation and supportive social contexts [45,46]. Such mechanisms are functionally consistent with BCW strategies like enablement and environmental restructuring, yet they remain fundamentally incompatible with coercion-based methods.

Concurrently, the mapped evidence reveals that contextual opportunity levers, particularly those necessitating organizational or systemic change, remain less consistently operationalized and evaluated. Advocacy for rethinking school-based PA interventions suggests that research should integrate with school systems, leverage existing provisions, and establish context-specific causal pathways [41–43]. This involves prioritizing changes in timetables, staff practices, and school culture instead of merely layering new program elements onto already overburdened systems. Policy-oriented frameworks reinforce this systemic logic. For instance, the ISPAH “Eight Investments” advocate for whole-of-school programs, active transport, urban design, and supportive public education as essential interventions to reshape opportunity structures and mitigate inequities [48]. Within this context, the mapped evidence reveals a trend where the BCW serves to define intervention components rather than to reshape delivery environments. Specifically, there is a missed opportunity to leverage the framework for creating the environmental conditions necessary to make PA more accessible and socially normative for young people.

While implementation and sustainability represent the most critical evidence gaps from a public health standpoint [21], these concerns should not overshadow the fundamental challenge of promoting this population’s PA. From a practical perspective, the absence of data on reach, adoption, fidelity, and adaptation makes it nearly impossible for schools, communities, and clinical services to determine whether BCW-informed approaches can be effectively scaled and maintained within routine environments [49]. This gap mirrors broader school health implementation research, where interventions may be efficacious but often fail at the “delivery” stage [50]. A pragmatic approach involves embedding a focused, decision-relevant implementation dataset into PA research. This includes tracking participation and reach, delivery fidelity, and indicators of sustainment potential, while maintaining a primary focus on PA as the effectiveness outcome. Actionable strategies derived from school-based evidence syntheses, such as professional training and the integration of interventions into school workflows, align closely with BCW-derived diagnoses of delivery-system barriers [50].

Digital and wearable components appeared frequently across the included studies, reflecting contemporary trends in PA promotion of children and adolescents. Although digital platforms can amplify key BCTs, they frequently introduce “feature creep” through an over-reliance on multiple functions with unclear mechanisms. When coupled with issues of unequal access and engagement decay, these factors underscore a critical concern frequently emphasized in digital behavior change syntheses [40,51]. The BCW mapping process offers a more rigorous approach in this context. Interventions become more interpretable and easier to optimize when digital features are explicitly justified against specific COM-B determinants, such as linking prompts to environmental opportunity and social features to social opportunity. In parallel, stronger reporting standards are needed so that digital and multi-component PA interventions can be replicated and meaningfully compared. The Template for Intervention Description and Replication (TIDieR) checklist, along with its specialized extensions for complex interventions, offers a practical framework to enhance reporting granularity without compromising the brevity required for academic publication [52,53].

Several priorities follow for the next phase of BCW work in PA promotion of children and adolescents. First, research must transcend simple “component mapping” to establish testable, developmentally plausible causal logic. For instance, if modelling and social support are selected to address social opportunity, evaluations should incorporate intermediate mediators such as perceived peer norms, social inclusion, and enjoyment—constructs empirically linked to adolescent PA participation [45]. Second, outcome measurement should align with the behavioral target and setting constraints. The school-based interventions may change activity patterns within school hours but not necessarily overall MVPA, so selecting outcomes that reflect intended mechanisms (e.g., participation, in-school MVPA, active transport) can reduce false negatives and improve interpretability [41]. Finally, intervention functions and techniques must be selected with explicit consideration of equity. Since this population’s PA is heavily patterned by socioeconomic status, gender, disability, and neighborhood opportunity, integrating meaningful youth co-design can minimize the discrepancy between intervention assumptions and the lived experiences of participants [19,54].

This review provides a structured map of BCW applications specifically within child and adolescent PA promotion, integrating evidence across settings and study designs. By tracing patterns across COM-B determinants, intervention functions and BCT content, the review highlights both a coherent “default toolkit” and where the field may be underleveraging opportunity-focused and system-compatible strategies.

A key limitation of this review is that it focused exclusively on studies explicitly using the BCW and/or COM-B framework. Grey literature, including government reports, conference abstracts, and theses/dissertations, was not included; therefore, some unpublished, practice-based, or policy-oriented applications of the BCW/COM-B model may have been missed. While this enabled a theory-specific synthesis, it may have excluded relevant studies using other theoretical or implementation frameworks that address similar constructs. In addition, the number of eligible studies was relatively limited, particularly across some populations and application contexts, which constrained the breadth of cross-study comparisons. Incomplete reporting of intervention content and mapping pathways in primary studies limited the granularity of cross-study comparisons, reflecting a broader reporting challenge in behavior change research.

## 5. Conclusion

While the application of the BCW framework in PA promotion of children and adolescents is expanding, research remains predominantly focused on developmental and feasibility stages. In cases where interventions were clearly specified, the prevailing approach integrated goal setting and action planning with instruction, demonstration, and social support. This clusters effectively around capability building and self-regulation. Significantly, the marked absence of coercive or restrictive functions reinforces the principle that PA promotion of this population must prioritize autonomy-supportive strategies over punitive or controlling methods. Moving forward, the BCW should be leveraged to address structural opportunity constraints within schools and communities more directly. Future research must specify mechanisms that align with developmental realities and incorporate a core set of implementation indicators to facilitate real-world adoption and sustainment, ensuring these efforts complement rather than displace the fundamental goal of improving this population’s PA.

## Supporting information

S1 File Search strategy. (DOCX)

S2 File BCIO to BCTTv1 mapping. (XLSX)

S3 File Detailed mappings of COM-B components and BCW intervention functions. (DOCX)

S4 File Implementation outcomes. (DOCX)

S5 File PRISMA-ScR checklist. (PDF)

S6 File Literature screening procedure. (XLSX)

S7 File Extracted data. (XLSX)

## References

[1] Bull FC, Al-Ansari SS, Biddle S, Borodulin K, Buman MP, Cardon G, et al. World Health Organization 2020 guidelines on physical activity and sedentary behaviour. Br J Sports Med. 2020;54(24):1451–62. doi: 10.1136/bjsports-2020-102955 33239350 PMC7719906

[2] Guthold R, Stevens GA, Riley LM, Bull FC. Global trends in insufficient physical activity among adolescents: a pooled analysis of 298 population-based surveys with 1·6 million participants. Lancet Child Adolesc Health. 2020;4(1):23–35. doi: 10.1016/S2352-4642(19)30323-2 31761562 PMC6919336

[3] Guo L, He H, Wang C. Are school-based behavioural interventions an effective strategy for improving physical activity and sedentary behaviour in children and adolescents? A meta-analysis. Front Pediatr. 2025;13:1532035. doi: 10.3389/fped.2025.1532035 40134909 PMC11933046

[4] Peng S, Khairani AZ, Yuan F, Uba AR, Yang X. Behavior change techniques in physical activity interventions targeting overweight and obese children and adolescents: a systematic review. Behav Sci (Basel). 2024;14(12):1143. doi: 10.3390/bs14121143 39767284 PMC11673257

[5] Tao R, Yang Y, Wilson M, Chang JR, Liu C, Sit CHP. Comparative effectiveness of physical activity interventions on cognitive functions in children and adolescents with Neurodevelopmental Disorders: a systematic review and network meta-analysis of randomized controlled trials. Int J Behav Nutr Phys Act. 2025;22(1):6. doi: 10.1186/s12966-024-01702-7 39806448 PMC11731537

[6] Telama R. Tracking of physical activity from childhood to adulthood: a review. Obes Facts. 2009;2(3):187–95. doi: 10.1159/000222244 20054224 PMC6516203

[7] Al-Walah MA, Donnelly M, Cunningham C, Heron N. Which behaviour change techniques are associated with interventions that increase physical activity in pre-school children? A systematic review. BMC Public Health. 2023;23(1):2013. doi: 10.1186/s12889-023-16885-0 37845721 PMC10580560

[8] Prestwich A, Webb TL, Conner M. Using theory to develop and test interventions to promote changes in health behaviour: evidence, issues, and recommendations. Curr Opin Psychol. 2015;5:1–5.

[9] Moore R, Edmondson L, Gregory M, Griffiths K, Freeman E. Barriers and facilitators to physical activity and further digital exercise intervention among inactive British adolescents in secondary schools: a qualitative study with physical education teachers. Front Public Health. 2023;11:1193669. doi: 10.3389/fpubh.2023.1193669 37346099 PMC10280726

[10] Craig P, Dieppe P, Macintyre S, Michie S, Nazareth I, Petticrew M, et al. Developing and evaluating complex interventions: the new Medical Research Council guidance. BMJ. 2008;337:a1655. doi: 10.1136/bmj.a1655 18824488 PMC2769032

[11] Skivington K, Matthews L, Simpson SA, Craig P, Baird J, Blazeby JM, et al. A new framework for developing and evaluating complex interventions: update of Medical Research Council guidance. BMJ. 2021;374:n2061. doi: 10.1136/bmj.n2061 34593508 PMC8482308

[12] Michie S, van Stralen MM, West R. The behaviour change wheel: a new method for characterising and designing behaviour change interventions. Implement Sci. 2011;6:42. doi: 10.1186/1748-5908-6-42 21513547 PMC3096582

[13] Michie S, Atkins L, West R. The behavior change wheel: a guide to designing interventions. London: Silverback Publishing; 2014.

[14] Couto N, Morgado V, Pereira T, Vitorino A, Bento T, Alves S, et al. Behavior change wheel as a tool to promote physical activity in online intervention: a case study. Front Psychol. 2025;16:1498351. doi: 10.3389/fpsyg.2025.1498351 40256440 PMC12007304

[15] Murphy K, Berk J, Muhwava-Mbabala L, Booley S, Harbron J, Ware L, et al. Using the COM-B model and behaviour change wheel to develop a theory and evidence-based intervention for women with gestational diabetes (IINDIAGO). BMC Public Health. 2023;23(1):894. doi: 10.1186/s12889-023-15586-y 37189143 PMC10186807

[16] Pelletier C, Ward K, Aird C, Jung M, Fox G, Pousette A, et al. Barriers and facilitators to physical activity in rural communities: using the behaviour change wheel to identify intervention functions. BMC Public Health. 2025;25(1):3315. doi: 10.1186/s12889-025-24672-2 41044481 PMC12495818

[17] Richardson M, Khouja CL, Sutcliffe K, Thomas J. Using the theoretical domains framework and the behavioural change wheel in an overarching synthesis of systematic reviews. BMJ Open. 2019;9(6):e024950. doi: 10.1136/bmjopen-2018-024950 31229999 PMC6596985

[18] McQuinn S, Belton S, Staines A, Sweeney MR. Co-design of a school-based physical activity intervention for adolescent females in a disadvantaged community: insights from the Girls Active Project (GAP). BMC Public Health. 2022;22(1):615. doi: 10.1186/s12889-022-12635-w 35351045 PMC8966245

[19] Mandoh M, Redfern J, Mihrshahi S, Cheng HL, Phongsavan P, Partridge SR. Shifting from tokenism to meaningful adolescent participation in research for obesity prevention: a systematic scoping review. Front Public Health. 2021;9:789535. doi: 10.3389/fpubh.2021.789535 35004591 PMC8734426

[20] Moore GF, Audrey S, Barker M, Bond L, Bonell C, Hardeman W, et al. Process evaluation of complex interventions: medical research council guidance. BMJ. 2015;350:h1258. doi: 10.1136/bmj.h1258 25791983 PMC4366184

[21] Proctor E, Silmere H, Raghavan R, Hovmand P, Aarons G, Bunger A, et al. Outcomes for implementation research: conceptual distinctions, measurement challenges, and research agenda. Adm Policy Ment Health. 2011;38(2):65–76. doi: 10.1007/s10488-010-0319-7 20957426 PMC3068522

[22] Aromataris E, Munn Z. JBI manual for evidence synthesis. Adelaide: Joanna Briggs Institute; 2020. doi: 10.46658/JBIMES-20-01

[23] Tricco AC, Lillie E, Zarin W, O’Brien KK, Colquhoun H, Levac D, et al. PRISMA extension for scoping reviews (PRISMA-ScR): checklist and explanation. Ann Intern Med. 2018;169(7):467–73. doi: 10.7326/M18-0850 30178033

[24] McHugh ML. Interrater reliability: the kappa statistic. Biochem Med (Zagreb). 2012;22(3):276–82. doi: 10.11613/bm.2012.031 23092060 PMC3900052

[25] Brennan C, O’Donoghue G, Keogh A, Rhodes RE, Matthews J. Developing an evidence- and theory-informed mother-daughter mHealth intervention prototype targeting physical activity in preteen girls of low socioeconomic position: multiphase co-design study. JMIR Pediatr Parent. 2025;8(e62795). 10.2196/62795 PMC11747544 39761561

[26] Caru M, Dandekar S, Gordon B, Conroy DE, Barb ED, Doerksen SE, et al. Implementing a behavioral physical activity program in children and adolescent survivors of childhood cancer: a pilot randomized controlled trial. J Behav Med. 2024;47(5):792–803. doi: 10.1007/s10865-024-00497-z 38735024

[27] Corr M, Murtagh E. “No one ever asked us”: a feasibility study assessing the co-creation of a physical activity programme with adolescent girls. Glob Health Promot. 2020;27(3):34–43. doi: 10.1177/1757975919853784 31232166

[28] Creaser AV, Bingham DD, Bennett HAJ, Costa S, Clemes SA. The development of a family-based wearable intervention using behaviour change and co-design approaches: move and connect. Public Health. 2023;217:54–64. doi: 10.1016/j.puhe.2023.01.018 36854251

[29] Faghy MA, Armstrong-Booth KE, Staples V, Duncan MJ, Roscoe CMP. Multi-component physical activity interventions in the UK must consider determinants of activity to increase effectiveness. J Funct Morphol Kinesiol. 2021;6(3):56. doi: 10.3390/jfmk6030056 34201440 PMC8293223

[30] Grimshaw SL, Taylor NF, Conyers R, Shields N. Promoting positive physical activity behaviors for children and adolescents undergoing acute cancer treatment: development of the CanMOVE intervention using the Behavior Change Wheel. Front Pediatr. 2022;10:980890. doi: 10.3389/fped.2022.980890 36313891 PMC9607881

[31] Maenhout L, Latomme J, Cardon G, Crombez G, Van Hove G, Compernolle S. Synergizing the behavior change wheel and a cocreative approach to design a physical activity intervention for adolescents and young adults with intellectual disabilities: development study. JMIR Form Res. 2024;8:e51693. doi: 10.2196/51693 38206648 PMC10811596

[32] Martin R, Murtagh EM. An intervention to improve the physical activity levels of children: design and rationale of the “Active Classrooms” cluster randomised controlled trial. Contemp Clin Trials. 2015;41:180–91. doi: 10.1016/j.cct.2015.01.019 25657052

[33] McDermott G, Brick NE, Shannon S, Fitzpatrick B, Taggart L. Barriers and facilitators of physical activity in adolescents with intellectual disabilities: an analysis informed by the COM-B model. J Appl Res Intellect Disabil. 2022;35(3):800–25. doi: 10.1111/jar.12985 35229409 PMC9305883

[34] Murtagh EM, Barnes AT, McMullen J, Morgan PJ. Mothers and teenage daughters walking to health: using the behaviour change wheel to develop an intervention to improve adolescent girls’ physical activity. Public Health. 2018;158:37–46. doi: 10.1016/j.puhe.2018.01.012 29544174

[35] Reedman SE, Jayan L, Boyd RN, Ziviani J, Elliott C, Sakzewski L. Descriptive contents analysis of ParticiPAte CP: a participation-focused intervention to promote physical activity participation in children with cerebral palsy. Disabil Rehabil. 2022;44(23):7167–77. doi: 10.1080/09638288.2021.1985636 34624202

[36] Taylor MJ, Arriscado D, Vlaev I, Taylor D, Gately P, Darzi A. Measuring perceived exercise capability and investigating its relationship with childhood obesity: a feasibility study. Int J Obes (Lond). 2016;40(1):34–8. doi: 10.1038/ijo.2015.210 26443341

[37] Wang H, Blake H, Chattopadhyay K. Development of a school-based intervention to increase physical activity levels among chinese children: a systematic iterative process based on behavior change wheel and theoretical domains framework. Front Public Health. 2021;9:610245. doi: 10.3389/fpubh.2021.610245 33987160 PMC8110714

[38] Wang H, Zhou Y, Blake H, Chattopadhyay K. School-based physical activity intervention: a qualitative process evaluation of a feasibility trial in Yangzhou, China. Int J Environ Res Public Health. 2022;19(2):1021. doi: 10.3390/ijerph19021021 35055842 PMC8775609

[39] Sivaramakrishnan H, Davis E, Obadimeji L, Valentine J, Wood F, Shetty V, et al. Behavior change techniques involved in physical activity interventions for children with chronic conditions: a systematic review. Ann Behav Med. 2024;58(8):527–38. doi: 10.1093/abm/kaae033 38917474

[40] Milne-Ives M, Homer SR, Andrade J, Meinert E. Potential associations between behavior change techniques and engagement with mobile health apps: a systematic review. Front Psychol. 2023;14:1227443. doi: 10.3389/fpsyg.2023.1227443 37794916 PMC10545861

[41] Jago R, Salway R, House D, Beets M, Lubans DR, Woods C, et al. Rethinking children’s physical activity interventions at school: a new context-specific approach. Front Public Health. 2023;11:1149883. doi: 10.3389/fpubh.2023.1149883 37124783 PMC10133698

[42] Porter A, Walker R, House D, Salway R, Dawson S, Ijaz S, et al. Physical activity interventions in European primary schools: a scoping review to create a framework for the design of tailored interventions in European countries. Front Public Health. 2024;12:1321167. doi: 10.3389/fpubh.2024.1321167 38389941 PMC10883314

[43] García Bengoechea E, Woods CB, Murtagh E, Grady C, Fabre N, Lhuisset L, et al. Rethinking schools as a setting for physical activity promotion in the 21st century–a position paper of the working group of the 2PASS 4Health Project. Quest. 2024;76(3):269–88. doi: 10.1080/00336297.2024.2318772

[44] Huéscar E, Moreno-Murcia JA, Domenech JF, Núñez JL. Effects of an autonomy-supportive physical activity program for compensatory care students during recess time. Front Psychol. 2020;10:3091. doi: 10.3389/fpsyg.2019.03091 32038432 PMC6992568

[45] Ntoumanis N, Moller AC. Self-determination theory informed research for promoting physical activity: contributions, debates, and future directions. Psychol Sport Exerc. 2025;80:102879. doi: 10.1016/j.psychsport.2025.102879 40383282

[46] Barbosa Cano D, Gomez-Baya D. Self-determination theory-based interventions to promote physical activity and sport in adolescents: a scoping review. Youth. 2025;5(3):98. doi: 10.3390/youth5030098

[47] Wanwan Z, Khairani AZ. The influence of perceived autonomy support on physical activity among high school students: the mediating roles of basic psychological needs. Behav Sci (Basel). 2025;15(4):536. doi: 10.3390/bs15040536 40282157 PMC12024411

[48] Milton K, Cavill N, Chalkley A, Foster C, Gomersall S, Hagstromer M, et al. Eight investments that work for physical activity. J Phys Act Health. 2021;18(6):625–30. doi: 10.1123/jpah.2021-0112 33984836

[49] Lane C, McCrabb S, Nathan N, Naylor P-J, Bauman A, Milat A, et al. How effective are physical activity interventions when they are scaled-up: a systematic review. Int J Behav Nutr Phys Act. 2021;18(1):16. doi: 10.1186/s12966-021-01080-4 33482837 PMC7821550

[50] Lee DC, O’Brien KM, McCrabb S, Wolfenden L, Tzelepis F, Barnes C, et al. Strategies for enhancing the implementation of school-based policies or practices targeting diet, physical activity, obesity, tobacco or alcohol use. Cochrane Database Syst Rev. 2024;12(12):CD011677. doi: 10.1002/14651858.CD011677.pub4 39665378 PMC11635919

[51] Hsu T-CC, Whelan P, Gandrup J, Armitage CJ, Cordingley L, McBeth J. Personalized interventions for behaviour change: a scoping review of just-in-time adaptive interventions. Br J Health Psychol. 2025;30(1):e12766. doi: 10.1111/bjhp.12766 39542743 PMC11583291

[52] Hoffmann TC, Glasziou PP, Boutron I, Milne R, Perera R, Moher D, et al. Better reporting of interventions: template for intervention description and replication (TIDieR) checklist and guide. BMJ. 2014;348:g1687. doi: 10.1136/bmj.g1687 24609605

[53] Signal N, Gomes E, Olsen S, Alder G. Enhancing the reporting quality of rehabilitation interventions through an extension of the Template for Intervention Description and Replication (TIDieR): the TIDieR-Rehab checklist and supplementary manual. BMJ Open. 2024;14(11):e084320. doi: 10.1136/bmjopen-2024-084320 39608992 PMC11603715

[54] Warraitch A, Wacker C, Biju S, Lee M, Bruce D, Curran P. Positive impacts of adolescent involvement in health research: an umbrella review. J Adolesc Health. 2024;75(2):218–30. 38597838 10.1016/j.jadohealth.2024.02.029

