# Supplementary material for: Applications of the Behavior Change Wheel in promoting physical activity among children and adolescents: A scoping review
Source: PLoS One. 2026 Jul 31;21(7):e0354697. doi: 10.1371/journal.pone.0354697 (PMC13426998; doi:10.1371/journal.pone.0354697)
Supplement: S1 File — (DOCX) [file pone.0354697.s001.docx]

# S1 Search Strategies for All Databases

## The following search strategies were applied across seven databases. Searches were conducted without language restrictions and covered the period from January 2011 to **5 August 2025**. Database-specific syntax and controlled vocabulary (e.g., MeSH, Emtree) were used where applicable.

## PubMed (109)

( "Behaviour Change Wheel"[tiab] OR "Behavior Change Wheel"[tiab] OR BCW[tiab] OR"COM-B"[tiab] OR "COM B"[tiab] OR (capability[tiab] AND opportunity[tiab] AND motivation[tiab] AND (behavio*[tiab] OR action*[tiab]))
) AND ("Motor Activity"[Mesh] OR "Exercise"[Mesh] OR "Exercise Therapy"[Mesh] OR "Sports"[Mesh] OR "Physical Fitness"[Mesh] OR "physical activ*"[tiab] OR exercis*[tiab] OR sport*[tiab] OR fitness[tiab] OR walk*[tiab] OR "motor activ*"[tiab]) AND ("Child"[Mesh] OR "Adolescent"[Mesh] OR pediatr*[tiab] OR child*[tiab] OR adolescen*[tiab] OR teen*[tiab] OR youth*[tiab] OR student*[tiab] OR school*[tiab]) AND ("2011/01/01"[Date - Publication] : "3000"[Date - Publication])

## Embase (132)

1. ('behaviour change wheel' OR 'behavior change wheel' OR bcw OR 'com-b' OR ((capability adj2 opportunity adj2 motivation) adj2 (behavio?r OR action$))).ti,ab.
2. exp physical activity/ OR exp exercise/ OR exp sport/ OR exp motor activity/ OR (physical activ* OR exercis* OR sport* OR walk* OR fitness).ti,ab.
3. exp child/ OR exp adolescent/ OR exp pediatrics/ OR (child* OR adolescen* OR teen* OR youth* OR student* OR school*).ti,ab.
4. 1 AND 2 AND 3
5. limit 4 to yr="2011 -Current"
6. limit 5 to human

## PsycINFO (56)

AB("behaviour change wheel" OR "behavior change wheel" OR BCW OR COM-B OR "COM B" OR (capability AND opportunity AND motivation AND (behavio* OR action*))) AND AB("physical activ*" OR exercis* OR sport* OR walk* OR fitness OR "motor activ*") AND AB(child* OR adolescen* OR teen* OR youth OR student* OR school* OR pediatr*)

Limiters: Publication Year from 2011; Human

## CINAHL (97)

AB("behaviour change wheel" OR "behavior change wheel" OR BCW OR COM-B OR "COM B" OR (capability AND opportunity AND motivation AND (behavio* OR action*))) AND AB("physical activ*" OR exercis* OR sport* OR walk* OR fitness OR "motor activ*") AND AB(child* OR adolescen* OR teen* OR youth OR student* OR school* OR pediatr*)

Limiters: Published 2011–present; Human

## Scopus (144)

(TITLE-ABS-KEY("behaviour change wheel" OR "behavior change wheel" OR BCW OR COM-B OR "COM B"
 OR (capability W/2 opportunity W/2 motivation W/2 (behavio* OR action*)))) AND (TITLE-ABS-KEY("physical activ*" OR exercis* OR sport* OR walk* OR fitness OR "motor activ*")) AND (TITLE-ABS-KEY(child* OR adolescen* OR teen* OR youth OR student* OR school* OR pediatr*)) AND (PUBYEAR > 2010)

## Web of Science Core Collection (160)

TS=("behaviour change wheel" OR "behavior change wheel" OR BCW OR COM-B OR "COM B" OR (capability NEAR/2 opportunity NEAR/2 motivation NEAR/2 (behavio* OR action*))) AND TS=("physical activ*" OR exercis* OR sport* OR walk* OR fitness OR "motor activ*") AND TS=(child* OR adolescen* OR teen* OR youth OR student* OR school* OR pediatr*)

Timespan: 2011–present; Indexes=SCI-EXPANDED, SSCI, A&HCI, ESCI

## Cochrane Library (45)

("behaviour change wheel" OR "behavior change wheel" OR BCW OR COM-B OR "COM B" OR (capability AND opportunity AND motivation AND (behavio* OR action*))):ti,ab,kw AND ("physical activ*" OR exercis* OR sport* OR walk* OR fitness OR "motor activ*"):ti,ab,kw AND (child* OR adolescen* OR teen* OR youth OR student* OR school* OR pediatr*):ti,ab,kw

Publication Year from 2011 to present
