# Supplementary material for: Applications of the Behavior Change Wheel in promoting physical activity among children and adolescents: A scoping review
Source: PLoS One. 2026 Jul 31;21(7):e0354697. doi: 10.1371/journal.pone.0354697 (PMC13426998; doi:10.1371/journal.pone.0354697)
Supplement: S3 File — (DOCX) [file pone.0354697.s003.docx]

**S1 Table.** Detailed mappings of COM-B components and BCW intervention functions

| **Study** | **COM-B: Psychological capability** | **COM-B: Physical capability** | **COM-B: Social opportunity** | **COM-B: Physical opportunity** | **COM-B: Reflective motivation** | **COM-B: Automatic motivation** | **BCW function: Education** | **BCW function: Persuasion** | **BCW function: Incentivisation** | **BCW function: Coercion** | **BCW function: Training** | **BCW function: Restriction** | **BCW function: Environmental restructuring** | **BCW function: Modelling** | **BCW function: Enablement** |
| --- | --- | --- | --- | --- | --- | --- | --- | --- | --- | --- | --- | --- | --- | --- | --- |
| Brennan et al., 2025 | Y | Y | Y | Y | Y | Y | Y | Y | NR | NR | Y | NR | Y | Y | Y |
| Caru et al., 2024 | Y | Y | Y | Y | Y | Y | NR | NR | NR | NR | NR | NR | NR | NR | NR |
| Corr & Murtagh, 2020 | Y | Y | Y | Y | Y | Y | Y | NR | NR | Y | Y | N | N | Y | Y |
| Creaser et al., 2023 | Y | NR | Y | Y | Y | Y | Y | Y | Y | NR | Y | N | Y | Y | NR |
| Faghy et al., 2021 | Y | Y | Y | Y | Y | Y | NR | NR | NR | NR | NR | NR | NR | NR | NR |
| Grimshaw et al., 2022 | Y | Y | Y | Y | Y | Y | Y | Y | Y | N | Y | Y | Y | Y | Y |
| Maenhout et al., 2024 | Y | NR | Y | NR | Y | NR | Y | Y | Y | N | N | N | Y | Y | Y |
| Martin & Murtagh, 2015 | Y | NR | NR | Y | Y | Y | Y | Y | NR | N | Y | N | Y | NR | Y |
| McDermott et al., 2022 | Y | Y | Y | Y | Y | Y | NR | NR | NR | N | NR | N | NR | NR | NR |
| McQuinn et al., 2022 | Y | NR | Y | Y | Y | Y | Y | Y | Y | N | Y | N | Y | Y | Y |
| Murtagh et al., 2018 | Y | Y | Y | Y | Y | Y | Y | Y | Y | NR | Y | NR | NR | Y | Y |
| Reedman et al., 2021 | Y | Y | Y | Y | Y | Y | Y | Y | NR | N | Y | N | Y | Y | Y |
| Taylor et al., 2015 | Y | Y | Y | Y | Y | Y | NR | NR | NR | N | NR | N | NR | NR | NR |
| Wang et al., 2021 | NR | NR | NR | NR | NR | NR | Y | Y | Y | N | Y | N | Y | Y | Y |
| Wang et al., 2022 | NR | NR | NR | NR | NR | NR | NR | NR | NR | NR | NR | NR | NR | NR | NR |

Notes: Y indicates that the item was reported in the study; NR indicates not reported.
