# Supplementary material for: Applications of the Behavior Change Wheel in promoting physical activity among children and adolescents: A scoping review
Source: PLoS One. 2026 Jul 31;21(7):e0354697. doi: 10.1371/journal.pone.0354697 (PMC13426998; doi:10.1371/journal.pone.0354697)
Supplement: S4 File — (DOCX) [file pone.0354697.s004.docx]

**S2 Table.** Implementation outcomes

| **Study** | **Implementation outcome – Acceptability** | **Implementation outcome – Adoption** | **Implementation outcome – Appropriateness** | **Implementation outcome – Feasibility** | **Implementation outcome – Fidelity** | **Implementation outcome – Implementation cost** | **Implementation outcome – Penetration/Reach** | **Implementation outcome – Sustainability** |
| --- | --- | --- | --- | --- | --- | --- | --- | --- |
| Brennan et al., 2025 | NR | NR | NR | NR | NR | NR | NR | NR |
| Caru et al., 2024 | Y | NR | NR | Y | NR | Y | NR | NR |
| Corr & Murtagh, 2020 | Y | NR | NR | Y | NR | NR | NR | NR |
| Creaser et al., 2023 | NR | NR | NR | NR | NR | NR | NR | NR |
| Faghy et al., 2021 | NR | NR | NR | NR | NR | NR | NR | NR |
| Grimshaw et al., 2022 | NR | NR | NR | NR | NR | NR | NR | NR |
| Maenhout et al., 2024 | NR | NR | NR | Y | NR | NR | NR | NR |
| Martin & Murtagh, 2015 | NR | NR | NR | NR | NR | NR | NR | NR |
| McDermott et al., 2022 | NR | NR | NR | NR | NR | NR | NR | NR |
| McQuinn et al., 2022 | NR | NR | NR | NR | NR | NR | NR | NR |
| Murtagh et al., 2018 | NR | NR | NR | NR | NR | NR | NR | NR |
| Reedman et al., 2021 | NR | NR | NR | NR | NR | NR | NR | NR |
| Taylor et al., 2015 | NR | NR | NR | NR | NR | NR | NR | NR |
| Wang et al., 2021 | NR | NR | NR | NR | NR | NR | NR | NR |
| Wang et al., 2022 | Y | NR | NR | NR | NR | NR | NR | NR |

Notes: Y indicates that the item was reported in the study; NR indicates not reported.
